# 

**Published:** 1902-09

**Authors:** 


					﻿Vol. XVIII.
SEPTEMBER, 1902.
No. 3.
TEXAS
Medical Journal.
Subscription, $i.oo a Year, in Advance.;'
Single Copies, io Cents.
PUBLISHED AT AU8TIN, TEXA8, BY
F. E. DANIEL, M. D.,
Proprietor.
Eastern Representative: John Guy Monlban, St. Paul Building, gSOYlTOUdWlL*. Mew
York Olty.
Entered at the Postoffice at Austin, Texas, as Second-class Mall Matter.
				

